# Sleep behavior and daily activity levels in people with metabolic syndrome: effect of 1 year of metformin treatment

**DOI:** 10.3389/fnut.2023.1240762

**Published:** 2023-09-27

**Authors:** Eleonora Bruno, Antonino Mulè, Letizia Galasso, Lucia Castelli, Ivan Baldassari, Andreina Oliverio, Elisabetta Venturelli, Franco Berrino, Angela Montaruli, Eliana Roveda, Patrizia Pasanisi

**Affiliations:** ^1^Department of Experimental Oncology, Fondazione IRCCS Istituto Nazionale dei Tumori di Milano, Milan, Italy; ^2^Department of Biomedical Sciences for Health, University of Milan, Milan, Italy; ^3^Department of Epidemiology and Data Science, S.C. Epidemiology and Prevention, Fondazione IRCCS Istituto Nazionale dei Tumori di Milano, Milan, Italy; ^4^IRCCS Ospedale Galeazzi-Sant'Ambrogio, Milan, Italy

**Keywords:** metformin, metabolic syndrome, lifestyle, sleep, rest-activity circadian rhythm

## Abstract

Impaired sleep and low daily activity levels increase the risk of developing metabolic syndrome (MS). Metformin (MET), an insulin sensitizer drug, is effective in regressing MS and has been recently studied as an adjuvant agent for managing sleep disorders. The present study aimed to assess whether 1,700 mg/day of MET treatment modifies sleep and daily activity levels in people with MS evaluated by Rest-Activity circadian Rhythm (RAR), which is the expression of 24 h of spontaneous activity parameters. A total of 133 subjects with MS, randomized into the MET (*n* = 65) or placebo (PLA, *n* = 68) group, underwent a clinical/anthropometric examination and carried out a continuous 7-day actigraphic monitoring to investigate sleep and RAR parameters at baseline and after 1 year of intervention. After 1 year of intervention, 105 subjects were analyzed. The MET group showed greater anthropometric and metabolic improvements compared with placebo, with a significant reduction in weight (*p* = 0.01), body mass index (*p* = 0.01), waist circumference (*p* = 0.03), and glucose (*p* < 0.001). With regard to sleep parameters, the MET group showed a significant increase in actual sleep time (*p* = 0.01) and sleep efficiency (*p* = 0.04) compared with placebo. There were no significant changes reported in the RAR parameters. Our study suggests that MET might be used as an adjuvant treatment for sleep disorders in people with MS.

## 1. Introduction

Metabolic syndrome (MS) is a cluster of cardiometabolic risk factors, such as abdominal obesity, high blood pressure, hyperglycemia, and dyslipidemia (1), that increase the risk of developing cardiovascular and other chronic age-related diseases (2, 3).

Genetic, metabolic, and environmental factors such as diet, physical activity, and sleep behavior play an essential role in the development of MS. Sleep behavior and daily activity levels evaluated by Rest-Activity circadian Rhythm (RAR), which is the expression of 24 h of spontaneous activity, can be considered reliable markers of the circadian timing system and may influence daily behaviors. They can change during the lifespan and can be considered indicators of a state of health or, otherwise, of disease (4). RAR alterations are related to various unhealthy conditions, such as neurological disorders (5), and also cancer (6, 7). Both insufficient sleep and circadian misalignment are also stressors to metabolic health and are associated with adverse health outcomes, including obesity (8, 9), hypertension (10, 11), hyperglycemia or insulin resistance (12, 13), and dyslipidemia (14).

Dietary habits and physical activity improve MS and sleep behavior. Specifically, sleeping for 7–8 h has been associated with better diet quality (15), higher intake of protein, vegetables, and fruits, and lower total fat (16). Furthermore, high levels of physical activity can decrease the risk of developing MS (17) or alteration in its related parameters (18) and produce beneficial effects on night-time sleep (19). In addition to physical activity, a more active daily routine and an increment in daily activity levels have been associated with better sleep quality (20). However, little attention has been given to the daily activity evaluated by RAR in people with MS (21, 22).

Metformin (MET) is an insulin sensitizer drug widely used to reduce insulin resistance and control glucose metabolism (23). It also reduces metabolic risk factors, prevents cardiovascular diseases in diabetics, and improves clinical outcomes in patients with heart failure. Recent data from a few preclinical studies (24, 25) support a relationship between MET treatment and the management of sleep disorders. One of the effects of MET is to promote the activation of AMPK, which stimulates fatty acid oxidation, promotes glucose transport and glycolysis, and inhibits gluconeogenesis. Consequently, it regulates insulin secretion and action, which are usually altered in concomitance with the presence of sleep disorders (26, 27). Kajbaf et al. (28) reported that diabetics treated with MET sleep longer and better compared with patients not treated with MET (28). In addition, it seems that MET is able to reduce sleep apnea in diabetes mellitus patients (29) and sleep disorders and daytime sleepiness in girls with polycystic ovarian syndrome (30). Xue et al. (31) showed that diabetics treated with MET have fewer sleep problems, but sleep less than the non-MET group (31). Conversely, Wiwanitkit and Wiwanitkit (29) reported that MET could induce insomnia and nightmares.

Up to now, no studies have investigated the effect of MET on sleep disorders in patients with MS.

The present study aimed to assess whether a 1-year treatment of 1,700 mg/day of MET can modify sleep parameters, daily activity levels, and the MS parameters in the volunteers with MS included in the Me.Me.Me. trial.

## 2. Materials and methods

### 2.1. Study design

Me.Me.Me. stands for Metabolic syndrome, Mediterranean diet, and Metformin. Detailed information about design, protocol, management system, and recruitment procedures has been previously described (32–36). In brief, “Me.Me.Me.” (ERC-AdG-2012 n.322752) is a phase III randomized controlled trial on people with MS for the primary prevention of age-related non-communicable chronic diseases (36). Me.Me.Me. was supported by an ERC grant and approved by the institutional review board and ethical committee of the Fondazione IRCCS Istituto Nazionale dei Tumori (approval number: INT 85/13). People aged 50–79 years presented at least three factors defining the MS such as waist circumference of >85 cm for women or >100 cm for men, fasting plasma glucose of ≥100 mg/dL, blood pressure of ≥130/85 mmHg, high-density lipoprotein (HDL) of <50 mg/dL for women and <40 mg/dL for men, and triglycerides of ≥150 mg/dL were eligible for the study. The design was 2 x 2 factorial, with 1,600 volunteers to be randomized into four groups of 400 each and allocated to the following treatment groups:

- Placebo alone (PLA);
- Metformin alone (MET);
- PLA + Mediterranean dietary intervention (DIET);
- MET + DIET.

At baseline, participants were asked to sign an informed consent form and attend an anthropometric/clinical visit, giving blood and urine samples. According to the definition of the International Diabetic Federation (1), after the baseline screening examinations, people with MS were included in the trial. Clinical visits and blood metabolic examinations were repeated at the end of the first year and annually for the whole duration of the study.

Among the Me.Me.Me volunteers, 133 (74 women and 59 men) participants, who did not take any drug/supplement to improve sleep, agreed to be also investigated about their sleep behavior and Rest-Activity circadian Rhythm (RAR) and signed an additional informed consent.

In the present study, the potential effect of MET on sleep parameters was assessed by comparing the MET vs. PLA group without taking into account the randomization by DIET.

We also analyzed MS parameters, sleep behavior, and RAR data in a 9% sample of the Me.Me.Me.

### 2.2. Measurements

Participants, at the baseline and the end of the first year of the study, were invited:

- to undergo an anthropometric (body composition, height, waist, and hip circumferences) and a clinical visit;
- to donate a fasting blood sample (glycemia, total cholesterol, HDL, LDL, triglycerides, liver enzymes, and creatinine);
- to wear the actigraph Motion Watch 8 (Cambridge Neurotechnology, Cambridge, UK) for 7 continuous days to collect RAR and sleep data.

#### 2.2.1. Sleep parameter assessment

Participants were instructed to wear the actigraph Motion Watch 8 and remove it when swimming or bathing. It is a triaxial accelerometer used to monitor the activity levels for 24 h for 7 consecutive days, expressed in activity counts (a.c.) and recorded every 60 s with a low actigraphic sensitivity threshold (20 counts per epoch) and validated for sleep monitoring (37). It was worn on the non-dominant hand's wrist, and participants received a daily diary to record bedtime, get-up time, and hours without wearing the actigraph. Actigraphic monitoring started during the first visit. During the monitoring, participants continued their usual activities. Sleep was evaluated with eight sleep parameters calculated automatically by the sleep analysis function of Motion Ware software 1.2.28 (CamNtech, Cambridge, United Kingdom) (38):

- Time in Bed: the total elapsed time between the “lights out” and “got up” times;
- Actual Sleep Time: the amount of sleep time, expressed in hours and minutes, between sleep start and sleep end;
- Actual Wake Time: the amount of awake time, expressed in hours and minutes, between sleep start and sleep end;
- Sleep Efficiency: the percentage of time in bed actually spent sleeping;
- Sleep Latency: the amount of time, expressed in hours and minutes, between sleep onset and retiring to bed;
- Mean Activity Score: the average activity score, expressed in activity counts, recorded between sleep start and sleep end;
- Immobile Time: total time expressed in percentage of no movement recorded between sleep start and sleep end;
- Fragmentation Index: an index that describes sleep fragmentation.

#### 2.2.2. Rest-activity circadian rhythm assessment

Rest-Activity circadian Rhythm (RAR) is the expression of 24 h of spontaneous activity for 7 consecutive days, and it can be objectively evaluated by actigraphy. On average, participants removed the actigraph once a day for ~30 min, and this period was not included in the data analysis.

The RAR was analyzed by the *single cosinor method* that describes the time course of 24-h activity using an oscillatory function: f(t) = M + A cos (ωt + ϕ) (39, 40). This function returns three rhythmometric parameters, depicting a parametric portrait of the activity rhythm for each subject: MESOR—Midline Estimating Statistic of Rhythm (M), Amplitude (A), Acrophase (ϕ). In brief, the MESOR is the rhythm-adjusted mean that approximates the arithmetical mean of the data for a given period. The amplitude determines half of the extent of the rhythmic variation in a cycle. The acrophase denotes the time interval within which the highest activity values are expected.

The method is based on the assumption that, since circadian rhythm can be considered as a smooth rhythm with added noise, a model based on a cosine curve (with a 24-h period) can be fitted by least squares to the data as an estimate of the pattern of the smooth rhythm. In the single cosinusoidal curve, the value of each point is a function of the average value of the variable investigated and estimated through the three parameters MESOR, amplitude, and acrophase (41).

If the *single cosinor analysis* gives back a statistically significant RAR, this means that the data are reliable for the rhythmometric analysis. Specifically, a data time series, expressed in activity counts and recorded every minute over the entire length of monitoring (7 days of 24 h each), was used to evaluate the subject's RAR and sleep using the *single cosinor method*.

Subsequently, to compare the groups, the RAR of each group, expressed by MESOR, amplitude, and acrophase, was evaluated using the *population-mean cosinor method*. When data are collected as a function of time on more than two individuals, the *population-mean cosinor method* allows to make inferences concerning a population rhythm (42). This method, applied to the rhythmometric parameters of each subject's circadian variables, evaluates the rhythmometric characteristics of the activity levels of the population analyzed.

### 2.3. Statistical analysis

#### 2.3.1. Sample size and power computation

The sample size was computed on patients with diabetes assuming an actual sleep time change as obtained from the study by Kajbaf et al. (28). Enrolling 60 patients per group would give ~80% power with two-sided alpha = 0.05, to detect a mean difference in the change of actual sleep time of ~30 min between the PLA and MET groups, and assuming an equal SD of 0.7. We enrolled ~65 patients per group to account for an anticipated drop-out rate of 5–10%.

#### 2.3.2. Analysis

Means and standard errors were calculated for each quantitative variable (anthropometric, metabolic, RAR, and sleep). The Shapiro–Wilk test was used to check the normality of the data distribution for all variables. Based on the results, unpaired parametric or non-parametric tests were used to investigate the difference at the baseline.

The main analysis aimed to test the effect of MET on sleep parameters after 1 year of treatment. The effect of MET on metabolic factors and RAR parameters was also evaluated. Univariate general linear models were used to perform the intention-to-treat (Δ of differences) analysis comparing the MET vs. PLA group. A two-level factor was used as the independent variable (MET vs. PLA*)* and MS, sleep, and RAR parameters were used as dependent variables; the analyses were adjusted for dietary intervention, age, sex, and values at baseline (covariates). As for the analyses on sleep and RAR parameters, we also controlled for Δ changes in MS condition.

An additional analysis was produced by the four randomization groups (Supplementary material).

The statistical analyses were performed using SPSS Statistics version 28 (IBM SPSS Statistics for Windows, Armonk, NY, USA: IBM Corp) and Rstudio statistics software (version 3.6.0), setting the statistical significance to *p* < 0.05. Power analysis was performed using G^*^Power—Universität Düsseldorf: Psychologie—HHU.

## 3. Results

### 3.1. Baseline

A total of 133 volunteers were randomized into the MET (*n* = 65) or PLA (*n* = 68) group. Table 1 describes the baseline characteristics of the whole study population and primary randomization group. The mean value of BMI was 31.7 ± 0.4, which corresponds to being obese. Although women differed from men in some sex-sensitive variables (height, weight, waist circumference, HDL, and fasting glucose) (data not shown), the anthropometric and metabolic characteristics were homogeneous in the MET and PLA groups (Table 1). Consistently, we did not observe any significant difference in the sleep parameters. In total, 29.3% of the population had a sleep efficiency higher than 85%, 15.8% showed an actual sleep time higher than 7 h, while 72.9% showed a sleep latency lower than 30 min [(cutoff value recommended by the National Sleep Foundation (43, 44)]. Men and women were comparable for all parameters, even if men presented slightly worse sleep quality and quantity than women (data not shown).

**Table 1:** Baseline anthropometric, metabolic, sleep, and RAR parameters in the total sample and by randomized group.

**Anthropometric and metabolic parameters**	**Total sample** ***N =* 133**	**PLA** ***N =* 68**	**MET** ***N =* 65**
Height *(cm)*	165.7 ± 0.8	165.8 ± 1.2	165.6 ± 0.9
Weight *(Kg)*	87.3 ± 1.4	88.5 ± 2.3	86.1 ± 1.6
BMI *(Kg/m^2^)*	31.7 ± 0.4	32.0 ± 0.6	31.5 ± 0.6
Waist circumference *(cm)*	101.8 ± 1.1	103.1 ± 1.7	100.6 ± 1.3
Systolic blood pressure *(mmHg)*	147.5 ± 1.4	147.0 ± 1.9	148.1 ± 2.1
Diastolic blood pressure *(mmHg)*	89.4 ± 0.8	89.6 ± 1.1	89.2 ± 1.2
Triglycerides *(mg/dL)*	121.4 ± 4.5	119.6 ± 6.1	123.7 ± 6.8
Fasting glucose *(mg/dL)*	102.3 ± 0.9	101.7 ± 1.3	103.0 ± 1.3
HDL *(mg/dL)*	54.6 ± 1.3	52.3 ± 1.6	56.8 ± 1.9
**Sleep parameters**			
Time in bed (*hh:mm*)	07:35 ± 00:05	07:28 ± 00:06	07:42 ± 00:07
Actual sleep time *(hh:mm)*	06:04 ± 00:04	06:00 ± 00:05	06:10 ± 00:07
Actual wake time *(hh:mm)*	01:01 ± 00:02	01:04 ± 00:02	01:00 ± 00:03
Sleep efficiency *(%)*	80.3 ± 0.6	79.8 ± 0.8	80.9 ± 0.9
Sleep latency *(hh:mm)*	00:22 ± 00:02	00:21 ± 00:02	00:23 ± 00:02
Immobile time *(%)*	83.6 ± 0.5	83.5 ± 0.7	83.7 ± 0.7
**Mean activity score** ***(a.c.)***	21.1 ± 1.2	21.4 ± 1.2	20.7 ± 2.1
**Fragmentation index**	34.8 ± 1.0	35.4 ± 1.5	34.1 ± 1.3
**RAR parameters**	**Total sample** ***N** =* **90**	**PLA** ***N** =* **49**	**MET** ***N** =* **41**
MESOR *(a.c.)*	219.2 ± 7.5	217.4 ± 10.4	221.3 ± 10.8
Amplitude *(a.c.)*	172.9 ± 6.5	167.6 ± 8.8	179.2 ± 9.7
Acrophase *(hh:mm)*	14:49 ± 00:08	14:48 ± 00:13	14:50 ± 00:11

Data are reported as mean ± se or percentage. BMI, body mass index; HDL, high-density lipoprotein; a.c., activity counts. PLA, placebo group; MET, Metformin group.

Only 90 of the 133 recruited subjects were found to have a statistically significant RAR, which means the presence of an individual rhythmic activity in 24 h.

After dividing the sample into four randomized groups (PLA, MET, PLA+DIET, and MET+DIET), no significant differences were found between the groups for all variables (Supplementary Table 1).

### 3.2 After 1-year intervention

During the first year of the study, participants in the PLA and MET groups consumed 10.6 and 10.7 bottles of the placebo or treatment, respectively (*p* = 0.75). After 1 year of intervention, 105 subjects (52 PLA and 53 MET) wore the actigraph and completed the anthropometric and metabolic examinations. Therefore, the adherence to the trial activities was ~76% for the PLA group and 81% for the MET group. Referring to the RAR analysis, statistically significant RAR was found in 61 of the 90 subjects, which means the presence of individual rhythmic activity in 24 h.

The before–after analysis showed that both groups significantly improved anthropometric and metabolic parameters, but people in the MET group showed changes with a greater impact on health (Table 2). The intention to treat analysis (Δ of differences) showed that the MET group lowered weight (*p* = 0.01), BMI (*p* = 0.01), waist circumference (*p* = 0.03), and glycemia (*p* < 0.001) significantly more than the placebo group (Table 2).

**Table 2:** Before–after and intention to treat analyses of MS, sleep, and RAR parameters in the sample randomized by the PLA and MET treatments.

**Anthropometric and metabolic parameters**	**PLA** ***N** =* **52**		**MET** ***N** =* **53**		Δ		
	**Baseline**	**Follow-up**	**Baseline**	**Follow-up**	**PLA**	**MET**	***p***
Height (cm)	165.9 ± 1.4	165.8 ± 1.4	165.4 ± 1.1	165.4 ± 1.1	−0.1 ± 0.0	0.0 ± 0.0	0.46
Weight (Kg)	89.4 ± 2.9	87.4 ± 2.9	85.0 ± 1.8	80.4 ± 1.6	–**2.0** **±0.5**	−**4.6** **±0.7**	**0.01**
BMI (Kg/m^2^)	32.3 ± 0.8	31.6 ± 0.8	30.7 ± 0.5	29.4 ± 0.5	–**0.7** **±0.2**	–**1.4** **±0.2**	**0.01**
Waist circumference (cm)	103.2 ± 2.0	100.5 ± 2.0	99.3 ± 1.4	95.1 ± 1.4	–**2.7** **±0.7**	–**4.2** **±0.7**	**0.03**
Systolic pressure (mmHg)	143.9 ± 2.1	144.6 ± 1.7	147.0 ± 2.3	144.4 ± 2.2	0.7 ± 1.8	−2.6 ± 2.1	0.41
Diastolic pressure (mmHg)	89.4 ± 1.3	88.8 ± 1.0	89.3 ±1.3	85.8 ± 1.2	−0.5 ± 1.3	−3.5 ± 1.3	0.09
Triglycerides (mg/dL)	127.6 ± 7.5	122.8 ± 7.9	109.8 ± 5.6	108.0 ± 7.0	−4.8 ± 5.7	−1.83 ± 5.3	0.83
Fasting glucose (mg/dL)	103.4 ± 1.5	102.3 ± 1.6	101.9 ± 1.6	97.6 ± 1.3	–**1.10** **±5.7**	–**4.32** **±1.1**	**< 0.001**
HDL (mg/dL)	52.2 ± 1.9	52.5 ±1.9	59.2 ± 2.1	59.8 ± 2.3	0.3 ± 1.0	0.7 ± 1.2	0.65
**Sleep parameters**							
Time in Bed (hh:mm)	07:29 ± 00:07	07:35 ± 00:07	07:36 ± 00:08	07:54 ± 00:08	00:06 ± 00:08	00:18 ± 00:09	0.09
Actual sleep time (hh:mm)	05:56 ± 00:07	05:58 ± 00:08	06:08 ± 00:08	06:30 ± 00:07	**00:02** **±00:07**	**00:22** **±00:08**	**0.01**
Actual wake time (hh:mm)	01:05 ± 00:04	01:06 ± 00:03	00:58 ± 00:03	01:00 ± 00:03	00:01 ± 00:04	00:02 ± 00:03	0.75
Sleep efficiency (%)	79.5 ± 0.9	78.5 ± 1.0	80.8 ± 1.1	81.9 ± 7.6	–**1.01** **±1.1**	**1.1** **±1.1**	**0.03**
Sleep latency (hh:mm)	00:21 ± 00:02	00:27 ± 00:02	00:23 ± 00:03	00:22 ± 00:02	00:06 ± 00:03	−00:01 ± 00:04	0.11
Immobile time (%)	83.4 ± 0.9	83.2 ± 1.0	83.6 ± 0.9	84.7 ± 0.7	−0.2 ± 1.0	1.1 ± 0.8	0.64
Mean activity score (a.c.)	22.4 ± 1.5	21.8 ± 1.7	20.5 ± 2.5	18.4 ± 1.1	−0.6 ± 1.9	−2.1 ± 2.5	0.14
Fragmentation index	35.7 ± 1.7	36.2 ± 2.0	34.2 ± 1.5	33.4 ± 1.6	0.5 ± 1.8	−0.7 ± 1.4	0.60
**RAR parameters**	**PLA** ***N** =* **32**		**MET** ***N** =* **29**		Δ		
	**Baseline**	**Follow-up**	**Baseline**	**Follow-up**	**PLA**	**MET**	***p***
MESOR (a.c.)	217.5 ± 13.7	208.1 ± 10.0	215.8 ± 13.4	196.4 ± 15.9	−9.5 ± 11.2	−19.4 ± 11.9	0.55
Amplitude (a.c.)	169.4 ± 11.6	169.7 ± 8.7	170.8 ± 10.9	164.4 ± 12.3	0.3 ± 9.5	−6.4 ± 10.3	0.63
Acrophase (hh:mm)	14:56 ± 00:12	**15:03** **±00:08**	14:52 ± 00:15	**14:36** **±00:12**	00:07 ± 00:10	−00:16 ± 00:09	0.10

Data are reported as mean ± se.

BMI, body mass index; HDL, high-density lipoprotein.

The bolded values correspond to a statistically significant comparison: *p* < 0.05. Analyses were adjusted for active dietary intervention, age, sex, and values at baseline. Sleep and RAR analyses were also adjusted for Δ change of MS condition. PLA, placebo group; MET, Metformin group.

The MET group also showed a good trend of improvement in most sleep parameters after 1 year of treatment. Conversely, the PLA group showed worsening sleep quality and quantity parameters (Table 2). The intention to treat analysis (Δ of differences) showed that the MET group significantly improved actual sleep time (*p* = 0.01) and sleep efficiency (*p* = 0.03) compared with PLA (Table 2).

An anticipated acrophase was found in the MET group compared with the PLA group. No other within and between groups differences were found, as well as in the intention to treat analysis we did not observe any differences (Table 2).

Analyzing the Δ changes of the four randomized groups after 1 year of intervention (Supplementary Table 2), the MET+DIET group showed changes with a greater impact on health compared with the other groups, with a significant reduction in weight (*p* < 0.001), BMI (*p* < 0.001), waist circumferences (*p* = 0.01), and fasting glucose (*p* < 0.01) compared with the PLA group. Regarding sleep, both the MET and MET+DIET groups increased the actual sleep time compared with the other two groups, even without significant results. Overall, the MET group presented an improvement, even though not significant, in the major part of sleep quantity and quality parameters compared with the other groups (Supplementary Table 2). The intention to treat analyses for RAR parameters did not show differences between the groups (Supplementary Table 2).

## 4. Discussion

Our results suggest that a 1-year treatment with 1,700 mg/day of MET significantly improves anthropometric parameters and glycemia in people with MS. The data also suggest that the MET group significantly improves actual sleep time and sleep efficiency compared with the PLA group.

Metformin reduces liver glucose production and absorption and increases the insulin sensitivity of target cells. It is indicated as an adjunct to diet, physical exercise, and lifestyle changes to improve glycemic and weight control in type 2 diabetes mellitus. Its main effects are observed in plasma glucose levels, but it also inhibits inflammation (in liver tissue, macrophages, vasculature, and adipocytes), increases energy metabolism by upregulating adaptive thermogenesis, inhibits lipid synthesis, and promotes fatty acid oxidation through the activation of AMPK (45).

Studies have shown that MET treatment is effective in reducing body weight and MS parameters (46–48). Few data are conversely reported on sleep behavior.

Previous studies have supported a protective association between sleep and metformin in people with type 2 diabetes or polycystic ovarian syndrome (29–31). About type 2 diabetes, a study on 387 outpatients found that patients treated with metformin slept for approximately 36 min longer and exhibited a 6.4% higher sleep efficiency compared with other treatments (28). Consistently, a cross-sectional study on 11,806 diabetic patients showed that those treated with non-metformin drugs were more likely to report difficulty falling and staying asleep at night compared with those on metformin monotherapy (31). Our results are consistent with these observations. In fact, we found an improvement of 22 min in actual sleep time and a significant increase in sleep efficiency in participants randomized into the MET group, independently from the dietary intervention, thus confirming a potential beneficial effect of this treatment also in people with MS. MS is an insulin-resistance condition that may be characterized by nocturia (due to poor glycemic control), obstructive sleep apnea, and hypoglycemic episodes that compromise sleep quality. Our hypothesis was that metformin, improving insulin resistance and, therefore, the MS parameters, effectively improves sleep. In the MET group we observed an improvement in sleep parameters. Therefore, as previously suggested, the mechanistic link between metformin and sleep is far from being exhaustively elucidated.

Regarding RAR, the effects of the intervention with metformin/placebo are less apparent. Indeed, people treated with MET or placebo did not differ or improve any of the RAR parameters. This result was rather predictable, as a change in daily routine is needed to modify the RAR, for example, by encouraging strategies aimed at increasing daily activity and decreasing sedentary behaviors. With this in mind, promoting structured physical activity interventions and actions aimed at promoting active habits in daily routines and lifestyles will be essential for future studies. Particularly sedentary lifestyles are increasingly associated with impaired sleep (49); thus, future interventions should aim to promote active lifestyles and reduce sedentary habits.

In this view, implementing a structured program of physical activity and lifestyle interventions in people with MS could be a complementary and reinforcing strategy, not only in order to improve RAR but also to improve sleep and modulate major health risk factors. Indeed, physical activity can not only change body composition and metabolic parameters leading to better health status (50, 51) but also can improve sleep behavior (52–54) in order to reduce the MS risk factors.

The current results suggest the hypothesis that metformin might be a new valid approach, together with a healthy lifestyle, in the management of MS and sleep parameters. Future studies that plan to associate the use of metformin with a structured program of physical activity in these subjects could be considered in order to improve the same parameters on which metformin acts. Moreover, a chronobiological approach that investigates the optimal timing of metformin intake could be useful to improve its effects on sleep and MS conditions.

An advantage of our study is that we objectively assessed sleep and RAR, using actigraphy, before and after 1 year of intervention. Reviews have shown that technology is preferred in trials because devices are typically portable, cost-effective, convenient, and accessible, allow for continual self-monitoring, and give the user a sense of control (55, 56).

The major limitation of this study is the small sample size. It is, however, interesting that, despite the small number, we observed an improvement in the metabolic and sleep parameters.

## Data availability statement

The raw data supporting the conclusions of this article will be made available by the authors, without undue reservation.

## Ethics statement

The studies involving humans were approved by Institutional Review Board and Ethical Committee of the Fondazione IRCCS Istituto Nazionale dei Tumori, Milan, Italy (approval number: INT 85/13). The studies were conducted in accordance with the local legislation and institutional requirements. The participants provided their written informed consent to participate in this study.

## Author contributions

FB, PP, EB, ER, and AMo: conceptualization. PP, EB, ER, and AMo: methodology. AMu and EB: formal analysis. FB, PP, ER, AMu, and EB: investigation. EB, AMu, AO, IB, LG, LC, and EV: resources. AMu, LG, and EB: data curation. EB, AMu, and LG: writing—original draft preparation. EB, AMu, PP, ER, LG, and FB: writing—reviewing and editing. FB, PP, ER, and AMo: supervision. PP: project administration. FB: funding acquisition. All authors have read and agreed to the published version of the manuscript.
