# Corrigendum: Sleep behavior and daily activity levels in people with metabolic syndrome: effect of 1 year of metformin treatment

**DOI:** 10.3389/fnut.2023.1327182

**Published:** 2023-11-13

**Authors:** Eleonora Bruno, Antonino Mulè, Letizia Galasso, Lucia Castelli, Ivan Baldassari, Andreina Oliverio, Elisabetta Venturelli, Franco Berrino, Angela Montaruli, Eliana Roveda, Patrizia Pasanisi

**Affiliations:** ^1^Department of Experimental Oncology, Fondazione IRCCS Istituto Nazionale dei Tumori di Milano, Milan, Italy; ^2^Department of Biomedical Sciences for Health, University of Milan, Milan, Italy; ^3^Department of Epidemiology and Data Science, S.C. Epidemiology and Prevention, Fondazione IRCCS Istituto Nazionale dei Tumori di Milano, Milan, Italy; ^4^IRCCS Ospedale Galeazzi-Sant'Ambrogio, Milan, Italy

**Keywords:** metformin, metabolic syndrome, lifestyle, sleep, rest-activity circadian rhythm

In the published article, there was an error in the Funding statement and funding from the Italian Ministry of Health was omitted. The correct Funding statement appears below.

## Funding

The Me.Me.Me. study was funded by an advanced grant from the European Research Council (ERC-AdG-2012 n. 322752). This research was also supported by Italian Ministry of Health “Ricerca Corrente” funds.

The authors apologize for this error and state that this does not change the scientific conclusions of the article in any way. The original article has been updated.

