# Supplementary material for: Sleep behavior and daily activity levels in people with metabolic syndrome: effect of 1 year of metformin treatment
Source: Front Nutr. 2023 Sep 27;10:1240762. doi: 10.3389/fnut.2023.1240762 (PMC10565813; doi:10.3389/fnut.2023.1240762)
Supplement: Supplementary file 1 [file Data_Sheet_1.docx]

**SUPPLEMENTARY MATERIAL**

**Table 1S.** Baseline anthropometric, metabolic, sleep and RAR parameters in the four groups.

| **ANTHROPOMETRIC**  **AND METABOLIC**  **PARAMETERS** | **PLA**  **N=26** | **PLA+DIET**  **N=38** | **MET**  **N=42** | **MET+DIET**  **N=27** |
| --- | --- | --- | --- | --- |
| **Height *(cm)*** | 167.0 ± 2.0 | 164.6 ± 1.1 | 165.0 ± 1.5 | 166.8 ± 1.8 |
| **Weight *(Kg)*** | 90.2 ± 3.7 | 84.8 ± 1.8 | 87.5 ± 3.1 | 88.0 ± 3.1 |
| **BMI *(Kg/m^2^)*** | 32.0 ± 0.7 | 31.4 ± 0.7 | 32.0 ± 0.9 | 31.6 ± 1.0 |
| **Waist circumference *(cm)*** | 104.4 ± 2.4 | 100.2 ± 1.2 | 102.2 ± 2.3 | 101.1 ± 2.6 |
| **Systolic blood pressure *(mmHg)*** | 153.7 ± 3.1 | 149.9 ± 2.8 | 142.8 ± 2.4 | 145.6 ± 3.1 |
| **Diastolic blood pressure *(mmHg)*** | 91.6 ± 1.9 | 90.5 ± 1.6 | 88.5 ± 1.4 | 87.3 ± 1.7 |
| **Triglycerides *(mg/dL)*** | 121.8 ± 9.2 | 113.5 ± 6.8 | 128.9 ± 9.7 | 120.5 ± 10.2 |
| **Fasting glucose *(mg/dL)*** | 104.8 ± 2.1 | 104.7 ± 1.9 | 99.8 ± 1.6 | 100.7 ± 1.8 |
| **HDL *(mg/dL)*** | 53.4 ± 3.4 | 60.6 ± 2.4 | 50.0 ± 1.5 | 54.4 ± 3.4 |
| **SLEEP**  **PARAMETERS** |  |  |  |  |
| **Time in Bed (*hh:mm*)** | 07:30 ± 00:08 | 07:42 ± 00:09 | 07:27 ± 00:09 | 07:42 ± 00:11 |
| **Actual Sleep Time *(hh:mm)*** | 05:59 ± 00:08 | 06:04 ± 00:09 | 05:56 ± 00:08 | 06:23 ± 00:10 |
| **Actual Wake Time *(hh:mm)*** | 01:04 ± 00:04 | 01:01 ± 00:03 | 01:01 ± 00:04 | 00:55 ± 00:04 |
| **Sleep Efficiency *(%)*** | 79.9 ± 1.1 | 79.2 ± 1.3 | 79.7 ± 1.2 | 83.2 ± 1.2 |
| **Sleep Latency *(hh:mm)*** | 00:19 ± 00:03 | 00:26 ± 00:04 | 00:24 ± 00:03 | 00:18 ± 00:03 |
| **Immobile time *(%)*** | 82.5 ± 1.2 | 82.6 ± 1.1 | 84.1 ± 1.0 | 85.3 ± 0.9 |
| **Mean Activity Score *(a.c.)*** | 21.2 ± 1.6 | 23.0 ± 3.4 | 21.5 ± 1.8 | 17.4 ± 1.8 |
| **Fragmentation Index** | 37.2 ± 2.6 | 36.2 ± 1.7 | 34.4 ± 1.9 | 31.1 ± 1.9 |
| **RAR**  **PARAMETERS** | **PLA**  **N=18** | **PLA+DIET**  **N=31** | **MET**  **N=25** | **MET+DIET**  **N=16** |
| **MESOR *(a.c.)*** | 220.1 ± 16.7 | 215.9 ± 13.5 | 217.5 ± 12.9 | 227.3 ± 19.3 |
| **Amplitude *(a.c.)*** | 167.9 ± 15.6 | 167.5 ± 10.8 | 167.6 ± 11.0 | 197.2 ± 17.8 |
| **Acrophase *(hh:mm)*** | 14:49 ± 00:25 | 14:47 ± 00:14 | 14:53 ± 00:17 | 14:45 ± 00:14 |

Data are reported as mean ± se or percentage. BMI= Body Mass Index; HDL= High Density Lipoprotein; a.c.=activity counts. PLA= placebo group; MET= Metformin group; DIET= Mediterranean dietary intervention

**Table 2S.** Before-after and intention to treat analyses of MS, sleep and RAR parameters in the sample randomazed by the four groups.

| **ANTHROPOMETRIC**  **AND METABOLIC**  **PARAMETERS** | **Δ of differences** | | | | |
| --- | --- | --- | --- | --- | --- |
|  | **PLA**  **N=17** | **PLA+DIET**  **N=35** | **MET**  **N=29** | **MET+DIET**  **N=24** | **p** |
| **Height *(cm)*** | -0.2 ± 0.1 | -0.0 ± 0.1 | -0.0 ± 0.0 | -0.1 ± 0.0 | 0.16 |
| **Weight *(Kg)*** | -1.7 ± 1.4 | -1.6 ± 0.4 | -3.6 ± 0.7 | -5.8 ± 1.3 | **<0.001** |
| **BMI *(Kg/m^2^)*** | -0.6 ± 0.5 | -0.6 ± 0.2 | -1.3 ± 0.2 | -2.1 ± 0.4 | **<0.001** |
| **Waist Circumference *(cm)*** | -2.7 ± 1.6 | -1.7 ± 0.5 | -3.0 ± 0.7 | -5.7 ± 1.3 | **0.001** |
| **Systolic Pressure *(mmHg)*** | -2.6 ± 2.0 | 3.0 ± 2.2 | -6.1 ± 2.4 | 1.5 ± 3.3 | 0.12 |
| **Diastolic Pressure *(mmHg)*** | -3.2 ± 2.7 | 1.3 ± 1.5 | -4.7 ± 1.6 | -2.0 ± 2.2 | 0.07 |
| **Triglycerides *(mg/dL)*** | 5.3 ± 10.9 | -9.1 ± 6.6 | 0.6 ± 6.1 | -4.8 ± 9.2 | 0.71 |
| **Fasting glucose *(mg/dL)*** | -0.9 ± 2.7 | -0.9 ±1.5 | -3.8 ± 1.8 | -5.0 ±1.3 | **<0.001** |
| **HDL *(mg/dL)*** | 1.5 ± 1.9 | -0.7 ± 1.1 | 1.0 ± 1.6 | 0.3 ± 1.7 | 0.77 |
| **SLEEP**  **PARAMETERS** |  | | | | |
| **Time in Bed *(hh:mm)*** | 00:11 ± 00:13 | 00:15 ± 00:10 | 00:19 ± 00:11 | -00:05 ± 00:27 | 0.25 |
| **Actual Sleep Time *(hh:mm)*** | -00:05 ± 00:14 | 00:06 ± 00:09 | 00:31 ± 00:11 | 00:11 ± 00:13 | 0.07 |
| **Actual Wake Time *(hh:mm)*** | -00:11 ± 00:05 | 00:07 ± 00:05 | 00:02 ± 00:05 | 00:01 ± 00:04 | 0.32 |
| **Sleep Efficiency *(%)*** | 1.08 ± 1.7 | -2.04 ± 1.4 | 2.9 ± 1.8 | -1.1 ± 0.9 | 0.12 |
| **Sleep Latency *(hh:mm)*** | 00:09 ± 00:05 | 00:04 ± 00:04 | -00:09 ± 00:05 | 00:09 ± 00:04 | 0.85 |
| **Immobile time *(%)*** | 2.6 ± 1.9 | -1.5 ± 1.2 | 1.5 ± 1.4 | 0.63 ± 0.8 | 0.34 |
| **Mean Activity Score *(a.c.)*** | -5.9 ± 2.1 | 2.0 ± 2.6 | -3.8 ± 4.5 | 0.01 ± 1.1 | 0.19 |
| **Fragmentation Index** | -3.3 ± 3.7 | 2.3 ± 2.0 | -0.5 ± 2.2 | -1.1 ± 1.5 | 0.90 |
| **RAR**  **PARAMETERS** | **Δ of differences** | | | | |
|  | **PLA**  **N=10** | **PLA+DIET**  **N=22** | **MET**  **N=16** | **MET+DIET**  **N=13** | **p** |
| **MESOR *(a.c.)*** | -26.4 ± 16.1 | -1.8 ± 14.6 | -24.4 ± 15.8 | -13.3 ± 18.5 | 0.78 |
| **Amplitude *(a.c.)*** | -13.8 ± 13.1 | 6.7 ± 12.4 | -1.0 ± 10.9 | -13.0 ± 18.9 | 0.48 |
| **Acrophase *(hh:mm)*** | 00:03 ± 00:01 | 00:09 ± 00:14 | -00:18 ± 00:11 | -00:12 ± 00:16 | 0.10 |

Data are reported as mean ± se. BMI = Body Mass Index; HDL = High Density Lipoprotein. The bolded values correspond to a statistically significant comparison: p<0.05. Analyses were adjusted for age, sex, and values at baseline. Sleep and RAR analysis were also adjusted for Δ change of MS condition. PLA= placebo group; MET= Metformin group; DIET= Mediterranean dietary intervention.
